# Innovation and challenges in obstructive sleep apnoea management: a critical review of diagnosis, treatment

**DOI:** 10.1136/bmjresp-2026-004473

**Published:** 2026-11-05

**Authors:** Supritha Senthilkumaran, Jolie Ghobrial, Ziyad Samara

**Affiliations:** 1 Medicine, Queen Mary University of London, London, England, UK; 2 Queen Mary University of London, London, England, UK

**Keywords:** Sleep apnoea, Telemedicine

## Abstract

Obstructive sleep apnoea (OSA) is a respiratory disorder of sleep caused by the complete or partial collapse and obstruction of the upper airways. This is due to a multitude of reasons, largely categorised as anatomical or neuromuscular abnormalities. This multifactorial pathophysiology leads to variable phenotypes of OSA. Currently, OSA remains largely underdiagnosed. It is associated with significant cardiovascular, metabolic and neurocognitive morbidity. Furthermore, current care pathways for OSA remain fragmented and largely centred on continuous positive airway pressure (CPAP).

This review explores the limitations of current OSA management pathways and highlights emerging diagnostic strategies, non-CPAP therapeutic options and the importance of integrated, multidisciplinary care models.

Despite CPAP being the gold standard therapy, its low adherence rates highlight the need for alternative treatments, including mandibular devices, transcutaneous electrical stimulation, upper airway surgery and emerging pharmacological agents targeting upper airways muscle tone.

Access to diagnostic services remains limited, with polysomnography mainly hindered by long waiting times. Innovations such as home sleep apnoea testing, wearable technologies and artificial intelligence-based triage systems offer promising pathways for earlier detection and monitoring; however, their integration into clinical care remains inconsistent.

Fragmented referral processes, a lack of standardised national pathways, and poor coordination between respiratory, ear, nose and throat and metabolic services continue to delay diagnosis and limit therapeutic success.

To improve outcomes, OSA care must shift towards phenotype-driven treatment within multidisciplinary teams. Embedding non-CPAP therapies into core care pathways and leveraging digital health innovations will be key to addressing the underdiagnosis and treatment dropout in OSA management.

## Introduction

 Obstructive sleep apnoea (OSA) is a highly prevalent and increasingly recognised sleep disorder that has been on the rise over the last few decades.^1^ It is reported to affect around 26% of the global adult population aged 21–70 years, with studies suggesting that up to one in four adults meet the criteria for at least mild OSA, while moderate to severe forms impact a significant minority. Additionally, OSA has been linked to substantial cardiovascular, metabolic and neurocognitive morbidities, such as myocardial infarction, hypertension, cardiac arrhythmias, stroke, type 2 diabetes and neurocognitive impairment, thereby placing a considerable burden on the National Health Service (NHS).^2^ Moderate to severe OSA is associated with an increased risk of all-cause mortality.^3^ In the UK alone, OSA is estimated to cost the NHS nearly £100 million annually due to heightened emergency visits, hospital admissions and long-term treatment requirements.^4^ These burden metrics highlight the urgent need for scalable diagnostic strategies and earlier risk stratification in primary care.

The most consistent risk factors of OSA are obesity and ageing, both of which are rising in prevalence worldwide and have further intensified the burden of OSA.^5 6^ Despite a significant health and economic burden, up to 80% of moderate to severe OSA cases remain undiagnosed, in part due to non-specific symptomatology (snoring, fatigue), gender and body mass index (BMI) diagnostic biases and limited access to specialist diagnostic services.^3 6–8^ This leads to a substantial downstream impact, despite advances in diagnostic methods and treatment technologies. OSA remains heavily underdiagnosed and insufficiently treated as a result. Polysomnography (PSG) remains the current diagnostic gold standard; however, new diagnostic methods are emerging to improve access and efficiency.^9^ Similarly, treatment remains dominated by continuous positive airway pressure (CPAP) despite challenges with adherence and tolerance. Other effective treatment methods are emerging but are underutilised or poorly integrated within current care pathways.^10^

This review explores the challenges of the current system and emerging innovations in the diagnosis and management of OSA, with particular focus on multidisciplinary and non-CPAP strategies.

## Methodology

A targeted literature search was conducted to evaluate current evidence on the diagnosis, phenotyping and management of OSA. The databases searched included PubMed, MEDLINE, Cochrane Library and relevant clinical guideline repositories. The search terms consisted of combinations of keywords including ‘obstructive sleep apnoea’, ‘OSA diagnosis’, ‘polysomnography’, ‘home sleep apnoea testing’, ‘wearable devices’, ‘artificial intelligence’, ‘biomarkers’, ‘OSA phenotyping’, ‘continuous positive airway pressure’, ‘oral appliances’, ‘surgical interventions’ and ‘metabolic therapies’. The search focused primarily on systematic reviews, original peer-reviewed research articles and clinical guidelines published between 2000 and 2026. UK clinical guidelines and healthcare frameworks were used as the primary reference point for evaluating current diagnostic and management pathways, reflecting the focus on service delivery and implementation within the UK healthcare system. International literature was also incorporated to provide broader perspectives on emerging technologies, alternative management strategies and developments in phenotype-driven OSA care. Additional sources, including NHS service evaluations and policy documents, were reviewed to explore healthcare delivery challenges, fragmented care pathways and opportunities for improved integration of OSA management. Sources were selected based on relevance to contemporary diagnostic approaches, emerging technologies and evolving treatment strategies, with evidence narratively synthesised to identify limitations in current practice and highlight innovations supporting personalised OSA management.

## Pathophysiology

The pathophysiology of OSA can be broadly characterised by repetitive episodic collapse of the upper airways during sleep, leading to intermittent hypoxia, fragmented sleep and systemic physiological stress.^11 12^ This collapse is primarily driven by a combination of anatomical and neuromuscular factors.^13^ Anatomical contributors include craniofacial abnormalities such as retrognathia, micrognathia and high-arched palate as well as soft tissue enlargement involving the tongue, tonsils and soft palate.^14^ This causes an increase in airway resistance and a decrease in airway pressure. Neuromuscular factors primarily involve pharyngeal muscle hypotonia during sleep, particularly during rapid eye movement (REM) sleep, which further compromises airway stability.^13^

Furthermore, obesity is a significant compounding factor in this process. Fat deposition in particular around the lateral pharyngeal walls and tongue base narrows the upper airway and increases collapsibility.^15 16^ Additionally, visceral fat affects respiratory mechanics by reducing lung volumes and tracheal traction, enhancing negative intrathoracic pressure during inspiration, which further destabilises the airway.^13^ These factors contribute to a more positive critical closing pressure (Pcrit), making the airway more susceptible to collapse even under normal breathing effort. Also, among apnoea driver mechanisms, ventilatory control instability, quantified by loop gain, has emerged as a targetable trait.^17^

This complex multifactorial mechanism, which underpins OSA, causes huge variabilities in disease presentation between individuals and therefore requires a more personalised approach to diagnosis and management rather than a one-size-fits-all treatment approach.^18^

## Diagnosis: current limitations and emerging innovations

Screening questionnaires are the preliminary step in OSA diagnosis and are often used for high-risk individuals to assess the need for referral for further testing.^19^ These tools are non-invasive, quick and cost-effective, aiding clinicians in being able to prioritise patients based on symptoms, severity and risk. Furthermore, it also ensures that patients do not go through unnecessary testing. The main questionnaires used to assess the risk of OSA include the Epworth Sleepiness Scale, STOP-Bang score (Snoring, Tiredness, Observed apnoeas, Pressure, BMI, Age, Neck size and Gender) and the Berlin Questionnaire.

Comorbid insomnia and sleep apnoea presents a distinct diagnostic challenge, as overlapping symptoms may obscure the typical clinical features of OSA.^20^ Affected patients often report fragmented sleep, persistent fatigue and cognitive impairment rather than classical excessive daytime sleepiness, while commonly used tools such as STOP-Bang and the Berlin Questionnaire are not designed to detect coexisting insomnia. Cardiorespiratory polygraphy may also underestimate OSA severity, as the absence of electroencephalographic monitoring limits accurate estimation of sleep time. Diagnostic assessment should, therefore, combine objective sleep testing with structured evaluation of insomnia symptoms and relevant comorbidities.

Timely and accurate diagnosis are crucial for managing OSA, but delays, access issues and diagnostic deficits often limit the current treatment regimens.^21^ As previously mentioned, PSG remains the current gold standard for diagnosis.^22^ PSG allows for a comprehensive sleep study, which analyses various physiological parameters during sleep. With the collected data, an apnoea-hypopnea index (AHI) is calculated, which helps determine the severity of OSA. Although PSG offers detailed insight into an individual’s sleep pattern and apnoeic events, it is resource-intensive, expensive and typically confined to specialist centres, thus leading to longer waiting times and geographical disparities in access to testing. Moreover, the PSG may not reflect a typical night’s sleep for the individual as they will be tested in an unfamiliar environment; this is known as the ‘first-night effect’.^23^

While AHI remains the conventional marker of disease severity, it fails to account for the heterogeneity of OSA.^24^ Clinical phenotyping has emerged as a framework to classify patients.^25^ It has become an increasingly valuable approach for stratifying patients with OSA based on symptom patterns, comorbidities and underlying pathophysiological mechanisms. While traditional classification of OSA has relied on AHI severity, newer models have emerged, which include phenotypes and endotypes of OSA; thus, taking into consideration the heterogeneous nature of OSA.

The AHI-based severity classification of OSA has well-recognised limitations that affect both diagnostic accuracy and clinical decision-making, which is a major shortcoming of current OSA classification systems. First is the night-to-night and physiologic variability. The AHI is influenced by body weight, sleeping position, age, alcohol, hypnotic use and fluid balance, so it can vary substantially across consecutive nights in the same patient.^26^ A single night polysomnogram may, therefore, misclassify disease severity and long-term risk, and measures such as time spent with oxyhaemoglobin saturation below 90% and nadir oxygen saturation may add prognostic information the AHI alone does not capture.

Another point is that AHI does not necessarily align with symptom burden; patients with similar AHI values can have markedly different degrees of daytime sleepiness, quality-of-life impairment or cardiovascular risk. Also, AHI conflates event types as the index incorporates obstructive, central and mixed events, which means further characterisation is needed before treatment decisions are made. Taken together, these limitations support a paradigm in which AHI is treated as one component of a broader clinical assessment—incorporating symptoms, oxygenation metrics, comorbidities and functional impact.

Recent cluster-analysis studies show that OSA patients can be grouped into discrete phenotypes with differing anthropometry, comorbidities and oxygen-desaturation patterns, reinforcing the need for phenotype-based rather than AHI-only assessment.^27^

A multidimensional framework can be used to map out OSA phenotyping. OSA pathophysiology exhibits multilayered determinants and clinical expression progressing from environmental factors to genetic predispositions, which further highlights the complexity of clinical and biological phenotyping of OSA and the importance of it for the management of OSA in every individual.^28^

Figure 1 broadly demonstrates the three main phenotypes, as the excessive daytime sleepiness phenotype, often linked to high cardiovascular risk, the disturbed sleep phenotype, characterised by insomnia-like symptoms and poor sleep quality and the minimally symptomatic phenotype, where patients may present with severe OSA on PSG despite subjective complaints.^29^ These differences highlight the limitations of solely relying on AHI for diagnosis and risk stratification of patients.^24^

**Figure 1:**
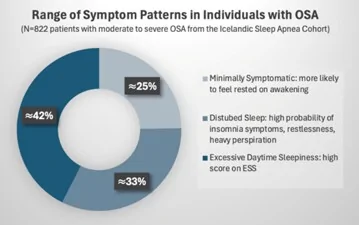
Clinical symptom phenotypes of OSA, including excessive daytime sleepiness, disturbed sleep and minimally symptomatic groups. The figure highlights the considerable variation in symptom burden among patients with OSA, demonstrating the limitations of relying on AHI alone. The data are based on 822 individuals from the Icelandic population.^29^ AHI, apnoea-hypopnoea index; ESS, Epworth Sleepiness Scale; OSA, obstructive sleep apnoea.

OSA can be classified into physiological endotypes, as well, which include upper airway collapsibility measured by Pcrit, pharyngeal dilator muscle responsiveness, arousal threshold and loop gain.^30 31^ Understanding both phenotype and endotype enables a more personalised approach to therapy.^32^ For instance, individuals with high loop gain may respond differently to therapies such as CPAP or oral appliances compared with those with normal loop gain.^17^

More recently, the Baveno classification has been proposed as an integrative model, combining symptoms and comorbidities into four clusters.^33^ For instance, a patient with severe OSA, but minimal symptoms yet, and high cardiovascular burden would be classified differently from one with moderate OSA but profound sleepiness. This framework moves beyond AHI, providing a clinically relevant tool for guiding treatment options, prognostics and prioritising patients for emerging non-CPAP therapies.^34^ However, current NHS pathways do not yet adopt this stratified model, reflecting the fragmented nature of OSA care, limiting the translation of clinical phenotyping into real-world practice.^35^

In the same context of a fragmented care pathway, improving diagnosis can be achieved with home sleep apnoea testing (HSAT).^36^ HSAT offers practical benefits, including improved accessibility, reduced cost and enhanced patient comfort, but results are less comprehensive than those attained using PSG testing. HSAT is a simplified version of the standard PSG conducted in a sleep laboratory; therefore, it records fewer physiological parameters. However, evidence suggests that in high-yield pretest probability patients, HSAT can yield similar outcomes to in-lab PSG testing. Although studies have not shown portable testing to be superior to PSG, the biggest advantage of HSAT is that it is done in the patient’s usual sleeping environment. The biggest drawback of HSAT is the often underestimation of severity in certain cases.^37^

More recently, wearable devices (namely, the WatchPAT, Fitbit, Sunrise device and Belun ring) and smartphone-based tools have introduced the possibility of large-scale, low-barrier screening.^38–41^ These devices incorporate various technologies that use sensors to measure snoring, oxygen saturation, heart rate, body position and mandibular movement. The sunrise devices use a small chin-mounted sensor to track mandibular movement as an indirect marker of respiratory effort.^41^ The Belun ring is also a newly developed wearable device, which records oxygen saturation, pulse rate and motion, using accelerometry to calculate total time and respiratory events.^39^ Recent studies have found that the Belun ring’s respiratory index correlated strongly with PSG’s AHI. In a cohort of hypertensive, high cardiovascular risk patients, the Belun ring showed good agreement with PSG.

This avenue has shown great promise, although it remains largely untested against the gold standard diagnostic method and therefore lacks standardised algorithms for clinical use.^42^ Wearable devices allow for more non-invasive patient-friendly alternatives to testing to traditional diagnostic methods and other HSAT methods, especially in ambulatory settings.

Moreover, research into salivary and blood biomarkers, including cysteine and malondialdehyde, indicates potential for early, simple, quick diagnosis and monitoring of OSA progression.^43–45^

A further innovation in OSA diagnostic tools includes the usage of artificial intelligence (AI) triage tools and cloud-based diagnostic platforms.^38 46^ This would enable real-time evaluation, scoring and risk stratification. Furthermore, it would allow for remote monitoring, which would be extremely valuable in primary care and underserved settings.

Overall, despite OSA being largely prevalent, it remains substantially underdiagnosed, with studies suggesting that up to 80% of moderate–severe cases go unrecognised.^7^ Several factors contribute to this diagnostic gap. The first symptomology is often non-specific fatigue, snoring or poor concentration, which can lead to misdiagnosis, especially in primary care.^8^ Second, there are recognised gender and BMI biases, with women and non-obese individuals being less likely to be referred for assessment, despite having clinically significant disease. These factors delay recognition and create inequalities in access to diagnostic services.

In the future, to diagnose OSA, a new, more efficient model should prioritise integration, use validated digital tools to expand access and align diagnostic strategies with the clinical phenotype and risk profile.^47^ Developing standardised, scalable diagnostic pathways will be key to reducing underdiagnosis and optimising resource utilisation.^37^

## Treatment

Current treatment for OSA has traditionally centred around the use of CPAP, which functions as a pneumatic splint by delivering constant positive airflow through nasal or oronasal masks.^48^ This airflow maintains upper airway patency and effectively prevents pharyngeal collapse during sleep. CPAP has consistently demonstrated high efficacy in reducing apnoeic events, improving oxygenation and alleviating daytime sleepiness across a wide range of OSA severities. It is very effective in preventing airway collapse during sleep and improving oxygenation. However, long-term adherence is poor, with up to 50% of patients discontinuing use due to discomfort, noise, air leaks, nasal congestion and disruption to their lifestyle.^49^

CPAP is often implemented as a universal solution, despite the wide variation in OSA phenotypes and severities. This further highlights the need for more personalised and tolerable alternatives. Oral appliances such as mandibular advancement devices (MADs) demonstrated in figure 2 are increasingly used in mild-to-moderate OSA or CPAP-intolerant patients.^50 51^

**Figure 2:**
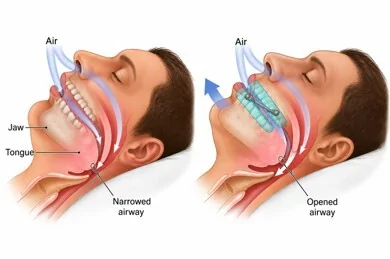
Mechanism of mandibular advancement therapy in OSA. The left panel demonstrates posterior displacement of the mandible and tongue during sleep, resulting in narrowing of the upper airway. The right panel illustrates forward positioning of the mandible, displacing the tongue anteriorly and increasing the calibre of the upper airway. This reduces the tendency for pharyngeal collapse during sleep and may improve airflow in appropriately selected patients.^50^ OSA, obstructive sleep apnoea.

Oral appliances work by advancing the mandible forward to enlarge the upper airways. While less invasive and more tolerable, their efficacy is limited in patients with obesity, craniofacial abnormalities or high AHI.^52^ Patients have also reported that long-term use can lead to jaw pain, dental malocclusion or temporomandibular joint dysfunction. Recent studies show that long-term MAD therapy remained effective after 5 years in more than 50% of patients with good patient adherence and symptomatic relief.^51^

Surgical options for OSA include uvulopalatopharyngoplasty (UPPP), maxillomandibular advancement (MMA) and hypoglossal nerve stimulation (HNS), each targeting specific anatomical causes of upper airway obstruction.^53^

UPPP involves surgical removal of soft tissues, the uvula, soft palate and tonsils, to increase upper airway patency.^53 54^ It is typically reserved for patients with soft tissue obstruction unresponsive to conservative therapies such as CPAP. However, outcomes vary, especially when deeper or multilevel airway collapse is present. Postoperative complications such as infection, velopharyngeal insufficiency and persistent symptoms are also concerns.

MMA repositions the maxilla and mandible forward to enlarge the airway and is mainly used in patients with severe OSA or craniofacial abnormalities (eg, retrognathia, micrognathia).^53^ While highly effective, it is invasive and requires a prolonged recovery.

HNS is a device-based intervention that stimulates the hypoglossal nerve directly to maintain tongue tone during sleep, reducing airway collapse.^53^ It is offered to CPAP-intolerant patients with moderate-to-severe OSA but is costly and unsuitable for patients with concentric airway collapse or high BMI.^55^

A new non-invasive intervention that targeted OSA patients with poor adherence to CPAP is the transcutaneous electrical stimulation (TESLA) in which an electrical current passes through skin to activate upper airway dilator muscles, opposite to HNS where the activation is direct.^56^

Other surgical interventions used adjunctively or in selected cases include nasal procedures (eg, septoplasty, turbinate reduction) to alleviate nasal obstruction, tongue-base reductions to address posterior airway narrowing and bariatric surgery, which targets obesity-related airway compromise by addressing the root cause of OSA severity.^57^

Emerging pharmacological therapies offer hope for targeted non-device-based treatments.^58^ The atomoxetine-oxybutynin combination has shown promise in enhancing upper airway muscle tone and reducing apnoeic events. Similarly, carbonic anhydrase inhibitors such as sulthiame have demonstrated reductions in AHI (particularly in patients with high loop gain).^59 60^ However, these treatments are still in the early clinical stages and require larger trials for validation.

Furthermore, for positional OSA, a new device has been recently approved for use by the Food and Drug Administration (FDA).^61^ This device, known as the Zzoma, is currently a prescription-only positional therapy designed to treat mild to moderate positional OSA. This device is worn around the upper torso in order to discourage back sleeping by making supine positions uncomfortable.^62^ Clinical evidence thus far suggests that the Zzoma positional therapy can reduce apnoeic events similar to CPAP in appropriately selected patients with positional OSA while offering greater comfort and fewer side effects since it avoids mask usage and high airway pressure.^61^ It has been proven to be particularly useful in patients who are CPAP intolerant for whom positional sleep apnoea is an established diagnosis. However, as with other positional devices, the effectiveness of the device is heavily dependent on correct device fit, patient compliance and accurate determination of positional dependency in sleep studies.

Moving forward, integrating phenotype-driven treatment models, telemonitoring tools and behavioural interventions is essential.^63^ A multidisciplinary team (MDT) approach, including respiratory physicians, ear, nose and throat (ENT) specialists, dentists, metabolic clinicians and behavioural health providers, can support personalised, effective and sustainable OSA management.^64^ National strategies should shift from CPAP-centric care to stratified, personalised care pathways that address both the patient’s symptoms and the root cause of the disease. As already mentioned, patients with different loop gain may respond differently to CPAP or MAD and also the level of loop gain can play a role in predicting success in certain types of surgery.^65^ In a very recent paper, phenotypic classification according to the PALM framework (pharyngeal collapsibility, arousal threshold, loop gain and muscle responsiveness) predicted postoperative outcomes in patients with OSA undergoing MMA.^66^

Drug-induced sleep endoscopy (DISE) is a relatively new technique in sleep medicine that helps in phenotyping OSA patients in many ways. For example, the MAD therapy guided through reproducible simulation of MAD effects during DISE demonstrated high positive predictive value allowing better selection of patients for such therapy.^67^

## Weight loss and metabolic interventions

As previously mentioned, obesity is a major factor contributing to OSA.^13^ Excess fat deposits around the upper airway cause narrowing, especially near the tongue base and lateral walls of the pharynx.^15^ This increases the chances of the airway collapsing. Additionally, visceral fat decreases lung volumes and tracheal traction, which reduces the caudal pull on the airway and further destabilises it.^13^ Weight loss can significantly improve OSA severity, particularly in patients with moderate to severe conditions.^68^ Research shows that even modest weight loss can lower AHI and reduce CPAP pressure needs. A 10% wt reduction is linked to a 26% decrease in respiratory disturbances. Although obesity is a primary cause of OSA, referrals to weight management services remain inconsistent, and these services often operate independently of respiratory or sleep clinics.

While bariatric surgery is included in many OSA treatment regimens, it is rarely accessed through sleep services directly. Bariatric surgery, including procedures such as sleeve gastrectomy or Roux-en-Y gastric bypass, has demonstrated significant improvements in AHI, BMI and cardiometabolic outcomes in patients with obesity-related OSA.^69^ It is particularly effective in cases of severe OSA with coexisting obesity, and in some instances can lead to complete resolution of the disorder.^69 70^ However, it is seldom accessed via sleep medicine pathways, and patient eligibility is often assessed independently by metabolic or surgical teams. This separation causes delays in access, underutilises a proven disease-modifying intervention and hampers continuity of care.

Metabolic medicine clinics often manage patients along separate referral pathways, leaving little coordination between teams. The same is true for patients receiving pharmacological weight loss agents such as tirzepatide, a dual glucose-dependent insulinotropic polypeptide /glucagon-like peptide-1 (GLP-1) receptor agonist, which is an FDA approved medication for obesity related OSA but not in any management pathways despite high prevalence of obesity among OSA patients.^71^ Other medications like the GLP-1 receptor agonists have shown potential not only to improve OSA severity but also to reduce cardiovascular risk.^72 73^

This disconnect highlights a systemic oversight in integrating weight loss interventions, both surgical and pharmacological, into the core treatment pathways for OSA. Despite obesity being a well-established primary driver of OSA, incorporating metabolic approaches into standard OSA care is clinically essential. Coordinated care between respiratory physicians and obesity management services, supported by MDT input, could significantly improve long-term outcomes and reduce reliance on CPAP.^74^ Future guidelines must incorporate weight loss and metabolic therapy as central elements of OSA management.

A summary of established and emerging therapeutic options is presented in table 1.

**Table 1:** Summary of established and emerging therapeutic options

Treatment	Advantages	Limitations	Target phenotype/endotype	Current evidence
Continuous positive airway pressure (CPAP)	Produces marked reductions in apnoeic events; improves oxygenation and daytime symptoms	Effectiveness is restricted by poor adherence related to discomfort, noise, air leak, nasal congestion and lifestyle disruption	Broadly applicable; increased upper airway collapsibility in multiple phenotypes	Established gold-standard therapy with consistent evidence of physiological and clinical benefit
Mandibular advancement devices (MADs)	Generally better tolerated than CPAP; can provide sustained symptomatic benefit with good adherence	Less effective in obesity, craniofacial abnormalities or high AHI; may cause jaw pain, dental malocclusion or TMJ dysfunction	Mild/ moderate disease with favourable mandibular/ craniofacial anatomy; CPAP-intolerance	Moderate; supported by several clinical studies but limited by variable response and less robust long-term data
Upper-airway surgery	Directly addresses the anatomical site of obstruction	Invasive, with variable outcomes and procedure-specific complications; some may require long recovery periods	Soft-palate, tonsillar, tongue base, nasal or craniofacial obstruction; lack of response to conservative measures	Low/ moderate; substantial heterogeneity between procedures and study designs
Hypoglossal nerve stimulation (HNS)	Maintains tongue tone to preserve airway patency without potential discomfort of positive airway pressure	Higher cost; anatomical and BMI suitability requirements limit broader clinical use	Mild/ moderate disease; tongue base obstruction with preserved neuromuscular response	Moderate; mainly based on selected patient cohorts
Domiciliary transcutaneous electrical stimulation (TESLA)	Directly targets upper airway muscle dysfunction without surgery; may provide a more affordable alternative to HNS for suitable responders	Uncertain long-term effectivity and durability; requires further studies; may cause skin discomfort and headache	Mild/ moderate disease due to impaired upper airway tone; CPAP intolerance	Early promising evidence; restricted by short follow-up period
Pharmacological therapy	Improves muscle tone; decreases the frequency of obstructive respiratory events	Larger trials are required to confirm efficacy, safety and appropriate patient selection	Impaired upper airway muscle responsiveness or ventilatory instability; high loop gain; CPAP-intolerance	Early stages; larger trials are needed for validation
Positional therapy	Better tolerability through avoidance of mask-related discomfort and pressurised airflow	Benefit depends on correct fit, compliance and confirmed positional dependency	Mild/ moderate supine-predominant disease	Promising evidence in appropriately selected patients
Lifestyle-based weight loss	Can reduce AHI and CPAP pressure requirements while improving wider cardiometabolic health	Weight loss may be difficult to achieve and maintain; OSA may persist despite improvement	Obesity-related upper airway narrowing, visceral adiposity, reduced lung volume and increased airway collapsibility	Well-supported as a disease-modifying component of OSA management
Bariatric surgery	Can produce marked improvements in AHI, BMI and cardiometabolic outcomes; can lead to OSA resolution	Invasive, with surgical risks and prolonged follow-up; remission is not guaranteed and access through sleep services is limited	Severe obesity; when substantial and durable weight loss is required	Strong evidence for improvement in obesity-related OSA
Pharmacological weight loss therapy	May improve AHI, BMI, cardiovascular risk and cardiometabolic markers through weight reduction	Relatively costly; not routinely incorporated into OSA pathways	Obesity-driven and metabolically-associated disease	Early promising evidence, but integration into routine OSA management remains limited

Treatments are compared according to their principal advantages, limitations, most relevant clinical phenotype or physiological endotype and the current strength of supporting evidence. Evidence descriptions are qualitative and reflect the maturity, consistency and generalisability of the literature discussed in this review rather than a formal GRADE assessment. Treatment selection should be individualised according to OSA severity, symptom burden, anatomical and physiological characteristics, comorbidities, treatment tolerance and patient preference.

AHI, apnoea-hypopnoea index; BMI, body mass index; GRADE, Grading of Recommendations Assessment, Development and Evaluation; HNS, hypoglossal nerve stimulation; OSA, obstructive sleep apnoea; TMJ, temporomandibular joint.

## Fragmented pathways and system-level barriers

Despite increasing awareness of OSA, the current diagnostic and treatment pathways remain disjointed and inconsistently applied across regions. Patients often begin their journey with primary care with non-specific symptoms such as fatigue and snoring.^3^ Following this initial presentation, the referral process varies from patient to patient, depending on regional protocols, with some patients directed to respiratory physicians, others to ENT specialists, and some to sleep clinics or even surgical services.^75^ This lack of standardisation on decisions of where to direct patients, whether to respiratory physicians, ENT specialists, sleep clinics or even bariatric services, is highly dependent on local protocols and service availability in national referral pathways of OSA, creating delays, duplicated assessments and even missed opportunities for early intervention especially in patients with high-risk profiles or comorbidities.

In the USA, analysis of a large national insurance claims dataset identified five common OSA diagnostic-treatment pathways, associated with meaningful differences in CPAP initiation rates, time to treatment and healthcare costs/resource utilisation.^76^ Drawing on insights from this analysis, adopting a clearly defined, unified pathway-based approach may help streamline access to therapy and limit avoidable expenditure.

In particular, access to diagnostic testing such as PSG, although considered the gold standard, remains constrained by long waiting times, regional disparities and limited capacity within specialised sleep centres.^77^ Alternative diagnostic strategies, including HSAT and wearable technologies, offer promising, scalable solutions but are not yet widely or consistently implemented across clinical settings.^36 38 40 42^ Likewise, AI-driven triage tools and cloud-based scoring platforms are beginning to emerge; however, their integration into existing national pathways is slow, inconsistent, and largely unregulated, thereby delaying their practical application and broader clinical impact.^38 41^

These inefficiencies also impact the development of novel therapies and the integration of research. Many patients would benefit from alternatives to CPAP treatments, such as weight loss, pharmacotherapy or surgical interventions, which are often neglected due to unclear referral.^77^ To improve outcomes, the OSA care pathway must be restructured into a cohesive, national framework supported by a robust MDT model. Shared digital systems, such as telemedicine, allow a multi-modal management approach whereby clinicians can remotely monitor patients and carry out actions, such as modifying CPAP settings as needed.^78^ This ultimately helps to improve patient care and increase compliance, especially in the case of CPAP. Furthermore, central triaging and clearer interdepartmental communication could transform patient experience and allow timely, evidence-based and holistic care.

## Future research

To reach the goal of better OSA management pathways, more research is needed to study the effects of surgery on OSA outcomes especially comparing the different surgical procedures.^79^

An example of such research that would contribute to OSA management is to reach a consensus on a scoring and classification system using DISE, to assess the role of DISE for titration of titratable OSA therapies such as upper airway stimulation therapy and to further explore the potential of DISE to optimise OSA treatment, by providing new insight into non-anatomical SDB pathophysiological factors and its relationship with upper airway configuration during DISE.^80 81^

Another area of research that will accelerate the personalised treatment is quantifying loop gain to embed it into routine phenotyping and treatment selection.^82^ On the other hand, more research needs to be done to assess long-term safety and cost-effectiveness of pharmacotherapy in OSA.

Finally, research on combining treatment methods is needed as some patients may need more than one mode whether sequential or at the same time.

## Conclusion

OSA is increasingly common and becoming a more complex clinical challenge due to its multifactorial pathophysiology and extensive systemic impact. Despite substantial research and technological advancement, the current management of OSA remains heavily dependent on CPAP. This often fails to address individual variability in disease presentations and pathophysiology.

One of the major limitations in OSA care is the fragmentation of diagnostic and therapeutic pathways. From the initial presentation in primary care to specialist referrals, there is significant inconsistency in how patients access services across different regions. This ultimately causes delays, duplicated assessments and gaps in follow-up. Additionally, there is a noticeable disconnect between sleep and metabolic services, especially related to the management of obesity in OSA.

Bariatric surgery and pharmacological agents such as Tirzepatide are increasingly utilised within metabolic pathways, yet they are seldom regarded as core treatment options within OSA guidelines. This dissonance neglects a significant opportunity for disease-modifying interventions. Likewise, innovative pharmacological agents targeting upper airway tone and ventilatory control remain insufficiently researched and underutilised due to a lack of integrated, phenotype-guided treatment models.

Diagnosis also suffers from inefficiencies. PSG remains the gold standard, but long waitlists and poor accessibility limit its utility in a population-level strategy. HSAT, wearable technologies and AI-assisted diagnostics are promising solutions but are not yet widely standardised or adopted in the current care pathways.

Improving OSA outcomes will require a shift from isolated, treatment-first approaches to comprehensive, system-level strategies. National care models should prioritise early diagnosis, patient-centred treatment selection and integration between respiratory, ENT and metabolic teams. The use of MDTs, digital tools for remote monitoring and stratified care pathways must become standard practice.

By embedding non-CPAP therapies, especially MAD,^83^ weight loss, surgery and later at a certain stage emerging pharmacological agents, into formal OSA pathways, care can become more personalised in accordance with the phenotype or endotype, sustainable and effective in reducing long-term disease burden.
